# Comparing Echocardiographic Modalities in Native-Valve Infective Endocarditis Due to Methicillin-Resistant Staphylococcus Aureus in People Who Inject Drugs

**DOI:** 10.7759/cureus.19372

**Published:** 2021-11-08

**Authors:** James Livesay, Tyler Coombes, Jared Spoons, Steven Dolacky, Mahmoud Shorman

**Affiliations:** 1 Department of Cardiovascular Medicine Fellowship, University of Tennessee Graduate School of Medicine, Knoxville, USA; 2 Department of Medicine, University of Tennessee Graduate School of Medicine, Knoxville, USA; 3 Department of Cardiology, University of Tennessee Graduate School of Medicine, Knoxville, USA; 4 Department of Infectious Diseases, University of Tennessee Graduate School of Medicine, Knoxville, USA

**Keywords:** methicillin-resistant staphylococcus aureus (mrsa), pwids (people who inject drugs), transesophageal echocardiography (tee), transthoracic echocardiogram, infective endocarditis

## Abstract

Background: Methicillin-resistant *Staphylococcus aureus* (MRSA) infective endocarditis (IE) is associated with high morbidity and mortality. Current IE guidelines recommend transesophageal echocardiogram (TEE) over transthoracic echocardiogram (TTE) to diagnose infective endocarditis. Management of IE in people who inject drugs (PWID) in many medical centers is mainly conservative with prolonged intravenous antibiotics. Cardiac valve replacement in these patients remains controversial, given the high risk of reinfection. This study’s purpose is to evaluate whether obtaining sequential TEE after TTE in PWID with MRSA native-valve IE changes the management plan in these patients.

Methods: A retrospective cohort of patients who are 18 years of age or older and inject drugs with definite MRSA IE between 2013 and 2019 were studied. Their echocardiographic reports and overall management plans were reviewed.

Results: One hundred and twenty-six patients met the inclusion criteria. TTE was performed in 121 patients and, of these patients, 69 (57%) had detectable valvular vegetations while 52 (43%) did not. Of the 52 patients with a negative TTE, 44 underwent TEE, 28 (53%) of which showed vegetation. A total of 18 (14%) patients underwent surgery. Of these, six (33%) patients had a positive TTE only, with no subsequent TEE. Ten (56%) patients had both a positive TTE and TEE, and two (11%) patients had a negative TTE but positive TEE.

Conclusion: In this retrospective cohort, obtaining a sequential TEE after a TTE in PWID with proven MRSA native IE by modified Duke’s criteria changed the management plan in two patients. The decision to perform a TEE in these patients needs to be individualized. Larger studies are needed to better evaluate the role of TEE in this patient population.

## Introduction

Infective endocarditis (IE) due to illicit injection drug use is becoming a major public health concern in the United States of America with increasing incidence from 27% in 2003 to 42% in 2013 [1,2]. IE in people who inject drugs (PWID) commonly affects the tricuspid valve with *Staphylococcus aureus* being the most commonly isolated pathogen [3,4]. The diagnosis of IE is one of the high clinical suspicions confirmed in conjunction with modified Duke criteria. Methicillin-resistant *Staphylococcus aureus* (MRSA) IE is associated with high morbidity and mortality [5]. As outlined in the modified Duke criteria, echocardiogram, while not required to make the diagnosis, is an essential diagnostic tool to adequately assess the location, extent, and functional impact of the infection, which is typically warranted in all cases of suspected and confirmed IE [3,6].

The *European Journal of Echocardiography* published in 2010 as well as the American College of Cardiology/American Heart Association and Infectious Disease Society of America (IDSA) recommend that all patients with *Staphylococcus aureus *bacteremia (SAB) undergo initial evaluation with a transthoracic echocardiogram (TTE), given its low cost, availability, safety, and diagnostic yield resulting in a sensitivity of 70% and specificity of 95% in native valves [3,7]. If the TTE is negative and suspicion remains high, current guidelines advise proceeding with a transesophageal echocardiogram (TEE), given its ability of increased spatial resolution, enhanced visualization of cardiac valves, and diagnostic yield with a sensitivity and specificity of over 95% [7,8]. TEE is also recommended by the IDSA in all cases of MRSA bacteremia [9].

Current treatment guidelines for MRSA IE consist of four to six weeks of intravenous antibiotics starting after the first negative blood culture and surgical evaluation. Indications for surgical intervention include large vegetation (>10 mm), ≥1 embolic event during the first two weeks of therapy, severe valvular insufficiency, valvular perforation or dehiscence, decompensated heart failure, perivalvular abscess, new heart block, and/or persistent bacteremia [9]. PWID despite having fewer comorbidities and being younger in age, due to recidivism, has been described as having a higher perioperative risk of adverse events and inferior mid-term survival compared to non-drug users, making the surgery decision more challenging [10]. In this cohort, we are evaluating the role of obtaining a TEE in addition to TTE and assessing if it changed the overall management in PWID diagnosed with MRSA IE.

## Materials and methods

This is a single-center, retrospective cohort, non-controlled study of 126 patients, which is performed at the University of Tennessee Medical Center (UTMC), a level I trauma center and academic hospital located in Knoxville, Tennessee. This study was approved by the UTMC Institutional Review Board, and the requirements for informed consent were waived. Inclusion criteria include age ≥ 18 years, hospitalization between January 2013 and January 2019, IE diagnosis per ICD9 and10 codes (421.1; I33.0), definite IE per the modified Duke criteria, positive blood cultures for MRSA, and PWID. Baseline patient characteristics were collected including gender, age discharge status, and antibiotic use.

Injection drug use was classified as intravenous drug abusers; people who received intravenous medications were not included in this cohort. Patients with prior valve replacement, indwelling vascular catheters, or pacemaker leads were excluded from this study. Each operative report from patients that underwent valvular intervention, defined as open valve replacement/repair or percutaneous intervention, was reviewed. Individual subjects were only included once in our cohort, and if one was eligible over multiple admissions, the first admission meeting the case definition was identified as the index admission or infection.

The primary outcome of the study was to evaluate whether obtaining a TEE, irrespective of TTE results, changed the overall management of MRSA IE patients. Change in management includes those patients who had vegetation identified on TTE, but pre- or intra-operative TEE identified other valvular involvement needing intervention or patients with a negative TTE but positive TEE requiring intervention.

Secondary endpoints include TTE image quality and valvular involvement. Each TTE report was reviewed for comments on image quality including good, poor, cannot rule out vegetation, or poor and cannot rule out vegetation. TTE reports were also reviewed for comments on patient cooperation such as combative and excessive movement during the study. Each TTE and TEE report was reviewed in detail to identify if one or more heart valves were involved.

## Results

One hundred and twenty-six patients met the inclusion criteria for this study. Fifty-six patients (44%) were males, and 70 (56%) were females. Of the 126 patients, 87 (69%) were discharged from the hospital, while 26 (21%) left against medical advice (AMA), and 13 (10%) expired (Table 1). Of the patients who expired, all 13 (100%) were transitioned to comfort care via family decision after discussions with palliative care. Reasons for transition to comfort care were mostly related to worsening septic shock in 10 patients (77%) along with aortic valve rupture in one patient (7.7%) who was deemed as not a surgical candidate, spinal cord infarction, progressive central nervous system (CNS) embolization in one patient (7.7%), and shock with disseminated intravascular coagulation (DIC) in one patient (7.7%). None of the patients who expired underwent valvular interventions. All patients in the study were treated with antibiotics for MRSA, including vancomycin, linezolid, daptomycin, or ceftaroline, by the infectious disease or medicine service.

**Table 1:** Characteristics of Patients PWID, People who inject drugs; ICU, intensive care unit; AMA, against medical advice.

Characteristics	Totals	Percentage
Total patients	126	
PWID	126	100%
ICU admission	63	50%
Mean age (years)	38	
Gender		
Male	56	44%
Female	70	56%
Discharge status		
Discharged	87	69%
Expired	13	10%
AMA	26	21%

**Figure 1:**
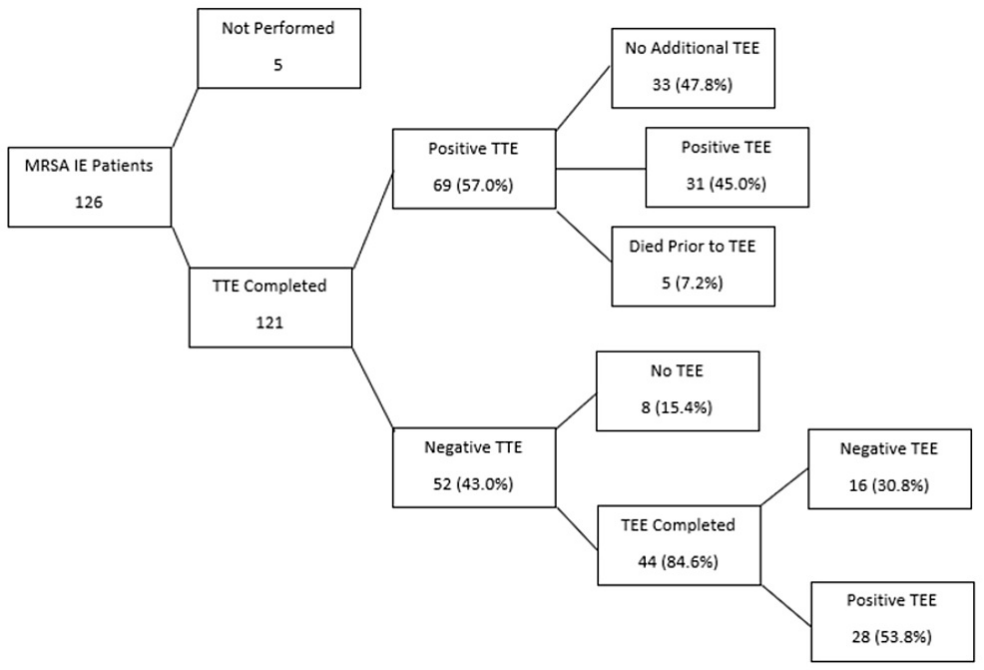
Flowchart of Echocardiographic Data MRSA, Methicillin-resistant *Staphylococcus aureus*; IE, infective endocarditis; TTE, transthoracic echocardiogram; TEE, transesophageal echocardiogram.

TTE was performed on 121 patients (96%), while five (4%) patients proceeded directly to TEE. Of those that preceded directly to TEE, two expired, one left AMA, and the other two had a high clinical suspicion secondary to septic pulmonary emboli and were later discharged. Of the 121 patients that underwent TTE, valvular vegetation was detected in 69 (57.0%), while no valvular vegetation was seen in 52 (43.0%). Of the 69 patients with a positive TTE, 31 subsequently underwent TEE, confirming IE. Out of the 52 patients with a negative TTE, 44 underwent TEE, of which 28 showed vegetation (54%) (Figure 1). Reasons TEE was deferred include inability to tolerate the procedure, leaving against medical advice prior to the procedure being performed, and death prior to the procedure being performed.

A total of 18 (14%) of the 126 patients underwent valvular intervention. Of these 18 patients, six (33%) had a positive TTE only, with no subsequent TEE. Ten (56%) had both a positive TTE and TEE, and two (11%) had a negative TTE but positive TEE. Reasons for intervention include worsening congestive heart failure (11%), recurrent septic cranial emboli (6%), splenic abscess (6%), shock with multi-organ failure (28%), persistent bacteremia (55%), and a large mobile mass (6%). In only two cases, the patient had a negative TTE and a positive TEE go on to surgical intervention (Table 2).

**Table 2:** Defining Factors for Surgical Intervention TTE, Transthoracic echocardiogram; TEE, transesophageal echocardiogram; HF, heart failure.

	Positive TTE Only	Positive TTE and TEE	Negative TTE with Positive TEE	Total (%)
Valvular intervention	6	10	2	18
Surgical intervention	5	5	2	12
Penumbra	1	5	0	6
Indications for surgical intervention				
Vegetation > 10 mm	0	1	0	1 (6%)
Embolic event	1	1	1	3 (17%)
Valvular insufficiency	0	2	0	2 (11%)
Valvular perforation	0	0	0	0
Decompensated HF	2	0	0	2 (11%)
Perivalvular abscess	0	0	0	0
New onset heart block	0	0	0	0
Persistent bacteremia	3	6	1	10 (55%)

Of the 121 patients that had a TTE performed, 89 (74%) were good-quality studies, 15 (12%) were poor-quality studies, 10 (8%) could not rule out vegetation, and seven (6%) were both poor-quality studies and could not rule out vegetation (Table 3). Of these 32 patients with a technically difficult TTE (poor quality, cannot rule out vegetation, or both), all underwent further evaluation with a TEE, and TEE was positive in 29 (91%). Of the patients with a negative TTE, 28 patients (54%) had a positive TEE in the setting of a negative TTE. The most common cardiac valve involved in our patient population was the tricuspid valve in 88 patients (70%), followed by the mitral valve in 20 (16%) patients (Table 4).

**Table 3:** Image Quality

TTE Image Quality		
Good	89	74%
Poor	15	12%
Cannot rule out vegetation	10	8%
Poor and cannot rule out vegetation	7	6%

**Table 4:** Valvular Involvement

Valvular Involvement		
Tricuspid	88	70%
Pulmonic	1	0.1%
Mitral	20	16%
Aortic	7	6%

## Discussion

With the ongoing opioid epidemic and increasing prevalence of injection drug use, the risks of MRSA bacteremia and IE are on the rise [11]. This study found that obtaining a TEE routinely after a TTE might not be cost-effective in this patient population of PWID with proven native-valve MRSA IE. TEE was done for 75 of the patients (60%) in our cohort, and a change in management resulting in a decision for valvular intervention was affected by TEE in only two of the cases.

In the diagnosis of IE, echocardiography remains the key imaging modality with higher sensitivity of TEE when compared to TTE in the identification of valvular lesions. This was also observed in our cohort with higher sensitivity of the TEE detecting vegetations in 54% of the patients with a negative TTE. However, compared to the TTE, TEE can be costly and resource-demanding, and it can be associated with procedural risks [12-15].

While the importance of echocardiogram in diagnosing IE must not be trivialized, the diagnosis can be made clinically via utilization of the other components of the modified Duke criteria with the incorporation of clinical, microbiologic, and laboratory findings and not relying solely on echocardiography or other imaging modalities [16,17]. In PWID, IE commonly involves the right-sided heart valves; it is usually associated with larger vegetation size and significantly increased risk of pulmonary thromboembolism, and it also carries a relatively lower mortality rate compared to left-sided endocarditis [18]. This was secondarily observed in our cohort with 70% of patients being diagnosed with tricuspid valve IE. The management of right-sided IE (RSIE) in PWID has traditionally been medical management with the appropriate antimicrobial therapy. Surgical management varies from one center to another but is usually reserved for patients who have uncontrolled sepsis or refractory heart failure despite appropriate antimicrobial therapy [19].

There has been some controversy previously about the optimal management of IE in PWID, with some studies showing poor long-term survival in this population despite undergoing surgery, usually due to the risk of non-compliance and recidivism, leading to significantly higher rates of reoperation in PWID compared to non-PWID [20,21]. This poses a clinical and logistical challenge in the management of IE in PWID, which has prompted the formation of multidisciplinary IE teams in many institutions to optimize service delivery [21,22].

In our cohort, 18 patients (14%) underwent valvular intervention, mainly due to persistent bacteremia and shock with multi-organ failure, despite being on appropriate antimicrobial therapy. Most of these patients (89%) had a positive TTE for IE, and two patients had a negative TTE but positive TEE. Of these two patients, the first patient underwent surgical intervention for persistent bacteremia and multiple pulmonary emboli. The initial TTE was negative for vegetation and was a good-quality study; however, the TEE did show a mass on the septal tricuspid leaflet. The second patient underwent surgical intervention due to septic shock and persistent cranial emboli. The TTE was noted to be a poor study due to patient movement.

In the MRSA treatment guidelines, the IDSA recommends performing echocardiography for all patients with SAB, preferably with TEE due to its higher sensitivity [9]. The evaluation and identification of IE on echocardiograms are based on the presence of one of these three major features: an oscillating intracardiac mass, abscess formation, or the presence of valve dehiscence of a prosthetic valve [16,23]. These are usually identified with good-quality TTE in native-valve RSIE, which was the case in most of our patient's cohort. The role of obtaining TEE after TTE is less clear [18]. Infectious disease physicians in a large academic healthcare system in Denver did not recommend proceeding to TEE in many cases of SAB as it would not have changed their management plan, which is usually a lengthy antimicrobial course, especially in the presence of concordant infectious focus like discitis or osteomyelitis and when there is no clinical evidence of IE complications [24].

All the patients in our cohort had infectious diseases consult, and TEE was done in 60% of the cases. Further research on the role of TEE among patients with SAB is needed to bridge the lack of concordance between current practice and the guidelines [25].

Some limitations of our study include its retrospective nature, small sample size, and single-center study in a region of the United States where intravenous drug use is above average. Larger multicenter studies are needed to further evaluate the study outcomes.

## Conclusions

Echocardiography remains the key imaging modality in patients suspected of having IE; however, if patients meet the modified Duke’s criteria for diagnosis of IE, invasive imaging may be able to be utilized more selectively. In our study, we evaluated the changes in management outcomes in patients who inject intravenous drugs, with proven MRSA native-valve endocarditis, and underwent TTE with subsequent TEE. We found that subsequent TEE only changed the management outcomes in two patients. This is an important finding as TEE is not without risks, which include but are not limited to damage to the esophagus and complications of anesthesia. Therefore, the decision to perform a TEE in this at-risk population needs to be individualized, and providers should consider whether the data from the TEE is likely to alter the management decisions prior to performing invasive imaging. Larger studies and prospective cohorts are needed to further evaluate the role of TEE in this patient population.
